# Surgical Treatment of Idiopathic spinal cord herniation: A Case Report under Neuromonitoring and Meta-analysis of 211 reviewed Cases

**DOI:** 10.1016/j.bas.2026.105958

**Published:** 2026-02-02

**Authors:** Umida Elmuradova, Ziad Omran, Stefanie Hammersen, Sven R. Kantelhardt, Ali Mulhem

**Affiliations:** a Vivantes Klinikum im Friedrichshain, Department of Neurosurgery, Berlin, Germany; b University of Oxford, Department for Continuing Education, Oxford, Northern Ireland, United Kingdom

**Keywords:** Idiopathic spinal cord herniation, Ventral spinal cord herniation, Thoracic spinal cord herniation, Anterior spinal cord herniation, Systematic review, Neuromonitoring

## Abstract

**Background:**

Idiopathic spinal cord herniation (ISCH) is a rare condition caused by a ventral or ventrolateral dural defect. Two surgical strategies are used: **non-closure (Group I),** consisting of reduction and adhesiolysis with or without enlargement of the defect, and **closure** (Group II), involving reduction followed by direct or indirect dural repair. This study compared these approaches.

**Methods:**

We reported a case of ISCH treated under neuromonitoring. We conducted a systematic review and meta-analysis including surgically treated cases confirmed by MRI or CT myelography, excluding traumatic, iatrogenic, and discogenic etiologies. Outcomes included neurological status at final follow-up, recurrence, and surgical complications.

**Results:**

A 50-year-old patient with progressive Brown–Séquard syndrome underwent surgery; neuromonitoring deterioration during attempted closure prompted conversion to non-closure. At the final follow-up, a clinical improvement without recurrence or complications was observed. Through the review, we identified 211 patients, including our case (mean age 50.99 ± 13.25 years; 58.7% female). Median follow-up was 24.16 months. In Group I, 53 improved, 4 were unchanged, and 2 worsened; in Group II, 114 improved, 27 were unchanged, and 9 worsened. Non-closure was associated with higher odds of improvement in the unadjusted analysis (POR 2.74, 95% CI 1.09–6.90, p = 0.032), but this association attenuated after adjustment (adjusted POR 2.53, 95% CI 0.69–9.31, p = 0.16). Complication rates were 3.38% vs 8.00% (OR 0.40, 95% CI 0.04–1.90); recurrence occurred once in each Group.

**Conclusions:**

Both strategies are comparable. The non-closure could be a better choice, since it requires less manipulation. Intraoperative neuromonitoring is a valuable decision-making tool in ISCH surgery.

## Highlights

• Idiopathic spinal cord herniation is rare, and treated surgically through two comparable approaches: non-closure and closure.
• The non-closure approach could provide a better choice through less spinal cord manipulation.
• Intraoperative neuromonitoring provides valuable guidance for surgical strategy.

## 1 Introduction

Idiopathic spinal cord herniation (ISCH) is a rare condition (Hajiabadi et al., 2023; Groen et al., 2019; Randhawa et al., 2020; Imagama et al., 2009; Nakamura et al., 2011; Sasani et al., 2009; Yamamoto et al., 2014; Summers et al., 2013; Hostettler et al., 2021; Prada et al., 2012; Nakashima et al., 2018; Ghosh et al., 2021; Aydin et al., 2011); the first published cases were reported by Wortzman in 1974 (Wortzman et al., 1974). Since the late 1990s, an increasing number of cases have been published (Sasani et al., 2009). ISCH is characterised by herniation of the spinal cord through a defect in the ventral or ventrolateral dura mater (Dix et al., 1998), most commonly in the thoracic spine, above all at T4/5 (Sasani et al., 2009). One published case describes ISCH occurring simultaneously in two separate zones at the intervertebral disc levels of T4–T5 and T5–T6 (Aydin et al., 2011). Matsumura et al. suggested that the thoracic spine's limited mobility and physiological curvature, which positions the spinal cord ventrally, likely contribute to a higher incidence of ventral thoracic spinal adhesions (Matsumura et al., 1996). Herniation of the spinal cord through the defect leads to incarceration and compression, which, if prolonged, can result in myelopathy (Nakamura et al., 2011). The literature frequently reports cases exhibiting dural duplication, dorsal arachnoid cysts, or both. A single case of a three-layer dura has been reported in the literature (Ellger et al., 2006). There are various theories about the pathogenesis of ISCH. Isu et al. postulated a specific sequence of events leading to spinal cord herniation and the formation of an extradural arachnoid cyst. They hypothesised that an intradural arachnoid cyst, on the dorsal side of the spinal cord, exerted chronic pressure on the surrounding dura. This sustained pressure caused thinning of the dura mater, and the weakened dura eventually developed a tear. Consequently, the spinal cord, along with the enlarged intradural arachnoid cyst, then herniated through this dural tear. This herniation occurred ventrally and into the extradural space, leading to the development of an extradural arachnoid cyst (Isu et al., 1991). According to Matsumura, herniation appears to require three successive factors: an abnormality in the dura mater, such as a defect, diverticulum, or duplication; adhesion between the spinal cord and the damaged dura; and continuous cerebrospinal fluid pressure that pushes the spinal cord outward from the subdural space. Several studies have sought to clarify the underlying pathophysiological mechanisms of this condition. Following a histological examination of herniated spinal cord tissue, Bartels published findings that support the hypothesis that idiopathic ventral herniation of the spinal cord is not an acquired entity. Instead, it's suggested to be the result of a minor developmental disorder occurring between 30 and 60 days of gestational age. Consequently, Bartels argues that the term" idiopathic herniation of the spinal cord" is a misnomer and should be replaced by, for example," a spinal dysraphism with a hamartomatous growth (Bartels et al., 2018). Shimizu, following a histopathological comparison of the ventral and dorsal dura in a patient with ISCH, reported degeneration limited to the ventral opening, which, although not specific to ISCH pathogenesis, suggested that the condition may have been a localised event occurring within an otherwise normal dural theca (Shimizu et al., 2019). Clinically, ISCH often presents in adult age with Brown-Séquard syndrome, but the clinical spectrum can range from mild pain to complete paralysis and bowel/bladder/erectile dysfunction (Sasani et al., 2009; Nakashima et al., 2018; Shin et al., 2010). The severity of neurological deficits appears to correlate with disease duration, progressing from monoparesis to Brown-Séquard syndrome and paraparesis (Hirose et al., 2023). In addition, it correlates with the spinal cord kink angle and the type of herniation based on localisation (Nakashima et al., 2018).

High-resolution and phase-contrast MRI, along with CT myelography, are considered the diagnostic modalities of choice. These imaging techniques are particularly useful for distinguishing idiopathic spinal cord herniation (ISCH) from dorsal arachnoid cysts or for identifying coexisting dorsal arachnoid cysts (Ghali et al., 2018).

To date, the literature has described both surgical and conservative treatment options, with some studies directly comparing their effectiveness. Ghosh published that conservative management is a viable option for patients with idiopathic spinal cord herniation, as it doesn't prevent symptomatic improvement (Ghosh et al., 2021). Ghali et al. recommended that treatment should be individualised, with surgical intervention favoured for patients with moderate to severe and/or progressive neurological symptoms (Ghali et al., 2018). Similarly, Nakamura et al. emphasised that surgical treatment should be considered before neurological deficits progress in patients with ISCH (Nakamura et al., 2011).

Two primary surgical approaches are employed (Sasani et al., 2009; Aydin et al., 2011).

1. The non-closure approach, which involves exposing the dural defect, reducing the herniation, performing adhesiolysis, and, with or without expanding the dural defect, preventing re-herniation and spinal cord incarceration (Sasani et al., 2009; Watanabe et al., 2001).
2. The closure approach, which entails reducing the herniation and either directly suturing the dural defect or using dural replacement materials, with or without sutures (Sasani et al., 2009; Nakazawa et al., 1993; Masuzawa et al., 1981).

Lui et al. reported a novel technique in which, in addition to duraplasty with a dural patch and glue, dentate ligament hitch stitches were applied to the dura. They suggest that this allows gentle dorsal migration of the spinal cord away from the dural defect and helps prevent recurrent spinal cord herniation. The technique was applied in two patients, both of whom achieved favourable clinical outcomes (Lui et al., 2018).

To date, there is no consensus on the optimal treatment for ISCH, and controversy persists over which of the aforementioned techniques is superior. Moreover, there is a lack of clear comparisons between these two approaches. Groen et al. suggest that dural extension may be preferable to anterior dural patching. However, their analysis included all anterior thoracic spinal cord herniations, not exclusively idiopathic cases (including iatrogenic cases) (Groen et al., 2019). We believe that ISCH requires distinct consideration and differentiation from other causes of spinal cord herniation, either caused by trauma, iatrogenic lesions or disco-bony pathologies, since SICH has possibly a unique pathogenesis (dural defect development) (Bartels et al., 2018), its chronic symptom progression compared to other types of spinal cord herniations, and potential differences in surgical outcomes. Therefore, this study aims to illustrate the course of ISCH through a case presentation and then compare dura non-closure and closure techniques in ISCH patients reported in the literature through a systematic review and meta-analysis, evaluating the impact on clinical state, postoperative complication rate, and recurrence risk.

## 2 Materials and methods

We present a case of ISCH treated at our centre, detailing the clinical course, imaging findings, surgical technique, and follow-up, and we have also conducted a PRISMA-guided systematic review. This research involved a comprehensive search of the PubMed database, encompassing both electronic and manual search methods, containing relevant keywords like" idiopathic spinal cord herniation"," ventral spinal cord herniation"," thoracic spinal cord herniation" and" anterior spinal cord herniation". The screening and data extraction were conducted in two stages: an initial screening of titles and abstracts, followed by a full-text analysis. The screening and data extraction were performed by a single reviewer (UE). The data was double-checked by this reviewer and subsequently validated through multiple random samples by another reviewer (AM).

### 2.1 Patients selection

We included English-language studies published on PubMed between 1974 and June 5, 2024, that reported on cases of patients with ISCH who underwent surgery.

Inclusion criteria.

• a diagnosis of ISCH confirmed by MRI and/or CT myelography
• surgical treatment
• no age restriction

Exclusion criteria.

• not published in English
• conservatively treated cases
• discogenic/bone disorder associated
• iatrogenic spinal cord herniation
• traumatic spinal cord herniation
• cervical spine cord herniation
• dorsal spine cord herniation
• Prior spine surgery
• major trauma in medical history
• spinal anomalies in medical history
• other neurological disorders in medical history

### 2.2 Patients groups

For analysis, cases of idiopathic spinal cord herniation were classified into closure and non-closure groups based on the intraoperative dural management strategy. Cases were assigned to the closure group when the dural defect at the site of herniation was directly closed after reduction of the herniated spinal cord, either by primary suturing or by patch augmentation aimed at restoring dural continuity and preventing re-herniation. In contrast, cases were categorised as non-closure when no attempt was made to close the dural defect; instead, the dural opening was intentionally enlarged or widened to relieve ventral tethering and allow free repositioning of the spinal cord without reconstructing the defect. This classification reflects the underlying surgical principle of either reestablishing dural closure or eliminating the constrictive effect of the defect by dural non-closure. Included cases were divided into two groups based on surgical approach: non-closure vs closure. The non-closure approach addresses the herniation and adhesiolysis, with or without expanding the defect. The closure approach primarily closes the defect, either directly or with a substitute.

Clinical outcome was categorised as improved, unchanged, or worsened based on postoperative neurological status as reported by the original studies, with improvement defined as any documented neurological recovery, unchanged status as no relevant postoperative neurological change, and worsening as any new or progressive neurological deficit. Postoperative complications were defined as any adverse events temporally related to the surgical procedure, such as cerebrospinal fluid leakage, epidural haematoma, syrinx, neurological deterioration, or the need for reoperation, as reported by the authors. Recurrence was defined as radiologically confirmed re-herniation of the spinal cord at the operated level during follow-up.

We compared dural non-closure (Group I) vs. dural closure (Group II) regarding symptom improvement at the end of follow-up (improved, unchanged, worsened), recurrence rate during follow-up confirmed by MRI and/or CT-myelography, and surgery-related complication rates.

### 2.3 Statistical analysis

Descriptive statistics are presented as mean ± standard deviation (SD) or median with interquartile range (IQR) for continuous variables, and as absolute numbers and percentages for categorical variables.

AM, a methodologist at the University of Oxford with expertise in systematic reviews and meta-analysis, performed statistical analyses. The primary outcome – ordinal neurological status at final follow-up (improved, unchanged, worsened) – was analysed using an **ordinal proportional odds regression model**, with surgical strategy (closure vs non-closure) as the main independent variable.

Secondary outcomes (recurrence and surgery-related complications) were analysed using **logistic regression**.

All analyses accounted for **clustering of patients within studies** using robust variance estimation and were **adjusted for potential confounders**, including age, baseline neurological severity, and duration of follow-up. A **sensitivity analysis** excluding cases with unclear surgical technique was additionally performed.

Statistical significance was defined as a two-sided p-value <0.05. All analyses were conducted using **Stata Statistical Software, Release 17** (StataCorp LLC, College Station, TX, USA).

## 3 Results

### 3.1 Case report

**Fig. 1:**
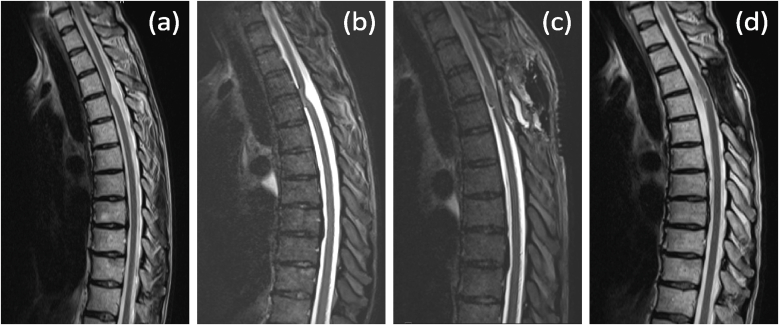
MRI of the thoracic spine in T2 sequence: a)demonstrates a kinked (C-shaped) spinal cord at the Th3/Th4 level 3 months before surgery; b) immediately preoperatively, showing signs of increased kinking of the spinal cord; c) directly postoperatively, confirming reduction of the hernia; d) at the 4-month follow-up, confirming resolution of the hernia.

A 50-year-old woman presented to our clinic with progressive numbness on the right side of her body below the level of the Th4 dermatome and motor coordination impairment in the left leg for approximately three months. These symptoms were consistent with Brown-Séquard syndrome. A thoracic spine MRI performed three months before presentation in our clinic revealed a kinked spinal cord (C-shaped) at the level of Th3/Th4 herniating ventrally to the left (Fig. 1a). The subarachnoid space dorsal to the kink appeared enlarged with flow voids, divided into two compartments by an abnormal dorsal subarachnoid septation.

**Fig. 2:**
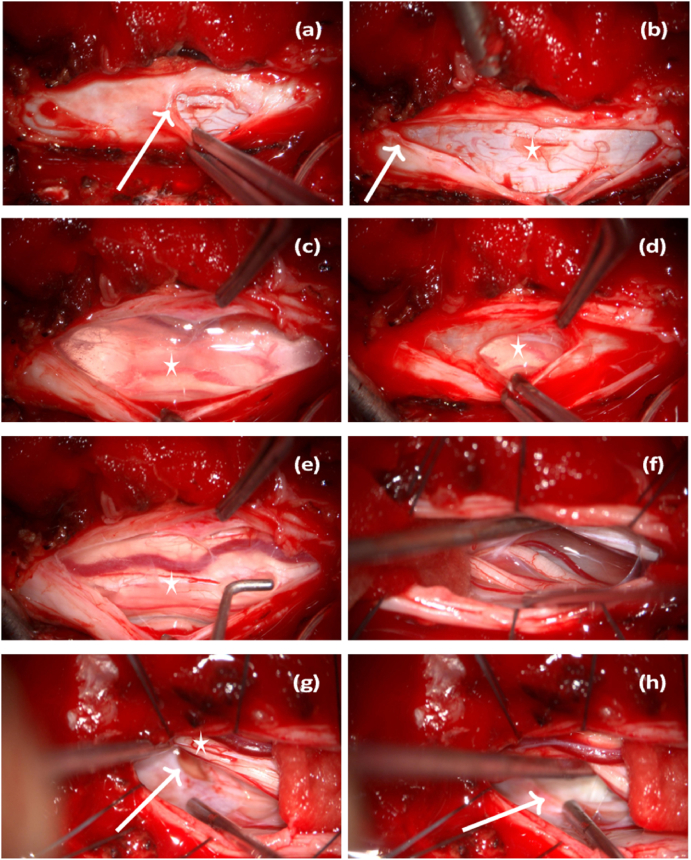
a) and b) outer dural layer of the dural duplication (indicated by arrow); b) inner dural layer of the dural duplication (marked with an asterisk); c) dorsal arachnoid cyst (marked with an asterisk); d) and e) ventral side of the spinal cord (marked with an asterisk); g) dorsolateral side of the spinal cord (marked with an asterisk); g) and h) dural defect (indicated by arrow).

At presentation, we performed a follow-up thoracic spine MRI, which showed progression of the myelopathic signal changes on the T2 sequence. (Fig. 1b). Based on the comprehensive findings, we determined that surgical intervention was necessary to reduce the spinal cord herniation. An operative procedure, which included intraoperative neuromonitoring, was performed involving a laminectomy at T3-T4 and removal of the spinous processes at T3-T4. Upon exposure, the dura appeared highly vascularised and a dural duplication was identified (Fig. 2a). This dural duplication was incised (Fig. 2b). An arachnoid cyst filled with cerebrospinal fluid emerged (Fig. 2c). The underlying spinal cord could be seen compressed (Fig. 2d). The arachnoid cyst was drained, allowing the spinal cord to partially shift dorsally. A dorsal subarachnoid septation was observed encircling the spinal cord. This septation was dissected and removed. Despite these manoeuvres, the spinal cord remained displaced to the left and could not be fully repositioned dorsally. Next, the ligamentum denticulatum was detached on both the left and right sides. The ventral aspect of the spinal cord was inspected (Fig. 2f), and the dural opening was extended cranially. A thorough inspection of the ventral aspect of the spinal cord was performed bilaterally. A defect measuring approximately 17 × 5 mm was identified in the inner layer of the dura, predominantly on the left side (Fig. 2g and h).

This defect allowed the herniation of the ventral spinal cord. The herniated spinal cord was carefully reduced dorsally under neuromonitoring (MEP and D-wave). Dural replacement material (Duragen) was applied bilaterally to seal the defect. The MEP demonstrated a reduced signal during this manoeuvre, prompting the decision to refrain from further ventral spinal cord manipulation and to remove the dural replacement material. The spinal cord now appeared symmetrical without lateral displacement. MEP and D-wave revealed a baseline signal. Directly postoperatively, the patient's neurological status remained unchanged. A direct postoperative MRI revealed complete reduction of the herniation (Fig. 1c). At the 4-month follow-up, the patient reported improvement in symptoms. The follow-up MRI confirmed complete resolution of the hernia (Fig. 1d). There was no recurrence or complications.

### 3.2 Systematic review

**Fig. 3:**
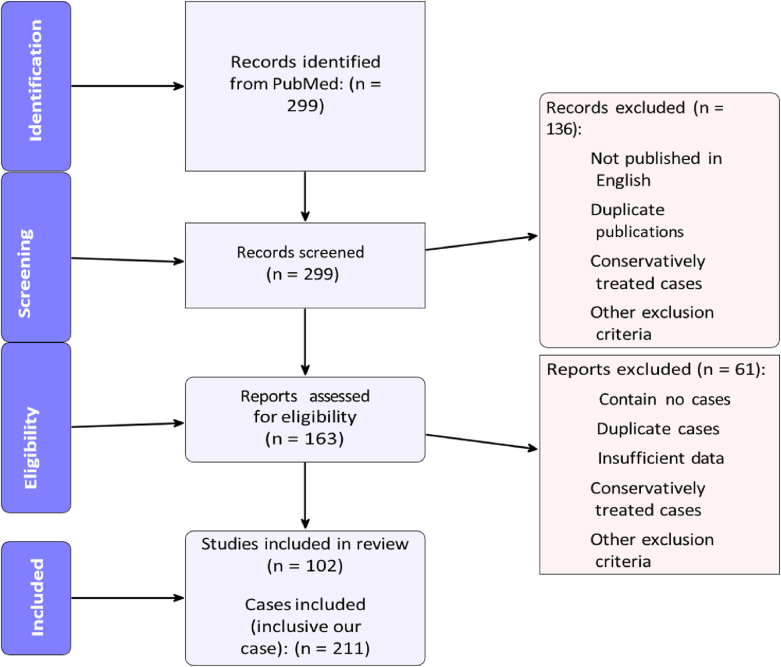
PRISMA flow diagram.

We identified 299 records published between November 1974 and June 5, 2024. Following an initial screening of titles and abstracts, 163 articles were selected for further review. Duplicate publications/cases and case reports with insufficient data were excluded. After full screening, 102 articles encompassing 210 ISCH cases met our inclusion criteria and were included in the study (the reference lists of included studies are provided in the Supplementary Material). Including our case, the total was 211. Fig. 3 presents the PRISMA flow diagram of the review.

The mean age was 50.99 ± 3.21 years, with 58.7% female and 41.3% male. The median follow-up was 12, with an IQR of 23. Group I (dura non-closure) included 59 patients, and group II (dural closure) 150. Due to a lack of specification regarding dural defect closure or expansion in their respective case reports, two patients could not be allocated to either group. Patients in both groups were comparable with respect to age, disease duration until surgery, and follow-up period (Table 1).

**Table 1:** Baseline characteristics between groups. BSS - Brown-Séquard Syndrome ± stool and urinary disturbances ± pain; MD - Motor deficits ± pain; SD - Sensory deficits ± pain; CMSD - Combined motor and sensory deficits ± Ataxia ± stool and urinary disturbances ± pain; P - Pain only/subjective symptoms.

Baseline characteristic	Group I	Group II
Total number of cases	59	150
Mean age in years	52,49	50,78
Sex in Percentage (male/female)	50/50	38/62
Mean disease duration in months	57,98	49,39
Preoperative diagnostic modality MRI/CT myelography/both	7/0/30	96/4/37
Neurological symptoms (severity) BSS/MD/SD/CMSD/P	25/16/0/12/0	43/22/9/75/0
Outcome improved/unchanged/worsened	53/4/2	114/27/9
Mean Follow-up period in months	38,14	20,81

#### 3.2.1 Clinical outcomes, clustering and sensitivity analyses

**Fig. 4:**
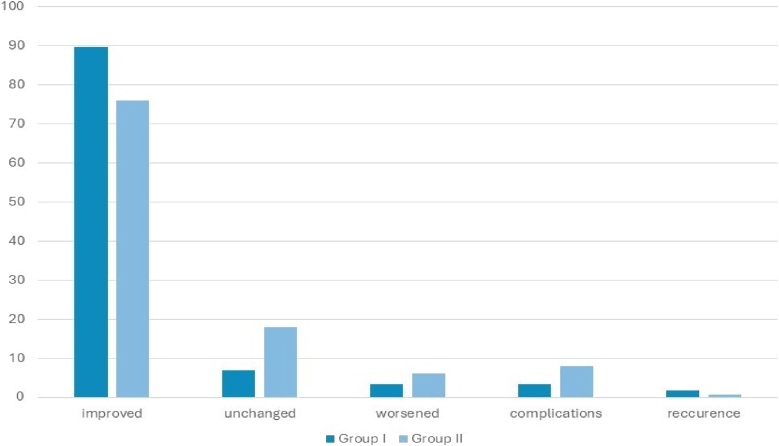
Results as percentages for each group.

In group I, 53 patients experienced symptom improvement, 4 had no change, and 2 worsened. In group II, 114 patients improved, 27 were unchanged, and 9 worsened (Fig. 4). The proportional odds ratio for symptom improvement in group I compared to group II was 2,74 (95 % CI: 1,16-7,58; statistically significant). However, after adjustment for follow-up duration, age, and baseline neurological severity, patients in the closure group had higher odds of a worse clinical outcome than those in the non-closure group (adjusted POR = **2.53**, 95% CI **0.69**–**9.31**; p = **0.16**), although this difference did not reach statistical significance.

To assess the robustness of our findings, we performed a sensitivity analysis restricted to cases in which the operative procedure was completely and clearly reported. In these analyses, the unadjusted proportional odds model demonstrated significantly higher odds of worse outcome in the closure group compared with the non-closure group (POR = **2.65**, 95% CI **1.03**–**7.78**; p = **0.045**). After adjustment for follow-up duration, age, and neurological severity, this association was attenuated and no longer statistically significant (adjusted POR = **2.53**, 95% CI **0.69**–**9.31**; p = **0.16**).

When accounting for clustering at the patient level using robust standard errors, the unadjusted proportional odds model showed higher odds of a worse outcome in the closure group than in the non-closure group (POR = **2.37**, 95% CI **0.72**–**7.79**). After adjustment for follow-up duration, age, and neurological severity, this association remained similar in magnitude but was not statistically significant (adjusted POR = **2.53**, 95% CI **0.71**–**9.06**). Table 2 summarises the results of the clinical outcome analyses.

**Table 2:** Clinical outcome with different statistical analyses.

Analysis Scenario	Adjustment	Clustering	POR	95% CI	p-value
**Primary (unadjusted)**	None	No	**2.74**	**1.09–6.90**	**0.032**
**Primary (adjusted)**	Follow-up, Age, Neuro severity	No	**2.53**	**0.69–9.31**	0.163
**Restricted sample (sensitivity)**	None	No	**2.65**	**1.03–7.78**	**0.045**
**Restricted + adjusted (sensitivity)**	Follow-up, Age, Neuro severity	No	**2.53**	**0.69–9.31**	0.163
**Cluster-robust**	None	Yes	**2.37**	**0.72–7.79**	–
**Cluster-robust + adjusted**	Follow-up, Age, Neuro severity	Yes	**2.53**	**0.71–9.06**	–

Two cases of recurrent herniation were observed, one in each group (OR = 2,59; 95 % CI from 0,18 to 36,42; statistically non-significant). The complication rate was 2 (3.38 %) in group I and 12 (8,00 %) in group II, with an odds ratio of 0,40 (95 % CI: 0,04-1,90), statistically non-significant (Fig. 4). Despite surgical complications, 50 % of cases in each group showed improvement. The worsening rate was also 50% in Group I and 41.7% in Group II (Table 3).

**Table 3:** Outcome of cases with complications.

	improved	unchanged	worsened
Group I	1	0	1
Group II	6	1	5

## 4 Discussion

Our findings indicate that the non-closure approach resulted in a comparable rate of improvement to the closure approach. While the non-closure approach also had a lower complication rate, this difference was not statistically significant. Regarding recurrence, both Group I (non-closure) and Group II (closure) experienced one case. However, because the non-closure group had 2.54 times fewer cases, its recurrence rate was proportionally higher, though this difference wasn't statistically significant. In summary, both complication and recurrence rates did not differ significantly between the two approaches. In our operation, intraoperative neuromonitoring (IONM) played a key role in decision-making. Numerous reports in the literature describe the use of IONM during surgery. For example, Hajiabadi et al. reported two cases in which surgery was performed under IONM guidance, with favourable outcomes. They conclude that IONM may help to confirm the efficacy of the intervention in future cases (Hajiabadi et al., 2023).

While various publications have sought to explain the optimal management of idiopathic spinal cord herniation, there's a notable absence of studies directly comparing surgical methods solely for this specific condition. Nakamura et al. reported that long-term surgical outcomes of dural defect enlargement for ISCH were stable and favourable; however, the study did not compare this approach with a dural closure technique (Nakamura et al., 2011). Another known but rare symptom of ISCH is cerebrospinal fluid hypotension-related headache in the upright position (Inoue et al., 2003; Maira et al., 2006). In such patients, the underlying cause is likely dural duplication, in which the residual outer layer of the dura mater prevents CSF leakage (Nakamura et al., 2011). Nakamura et al. recommended that enlargement of the dural defect should be considered for ISCH cases caused by dural duplication (Nakamura et al., 2011). In our study, we identified 40 cases with reported dural duplication and one case with a three-layer dura configuration. Of these, 30 were treated using a non-closure approach, while the remaining 11 underwent a closure-based technique.

Our findings align with the meta-analysis by Groen et al. (2019), which similarly demonstrated high improvement rates following surgical intervention (Groen et al., 2019). Both studies found that widening the dural defect yielded better outcomes compared to dural closure. Notably, our study focused exclusively on idiopathic spinal cord herniations.

Postoperative outcomes are influenced by various factors, including the severity and duration of preoperative symptoms, the location of the dural defect, and the patient's age. Patients with more severe neurological deficits tend to have poorer outcomes. For example, patients initially presenting with Brown-Séquard syndrome showed significantly greater symptom improvement than those presenting with spastic paralysis (Sasani et al., 2009). Patients who undergo surgery earlier in the course of their disease tend to have better outcomes than those who wait longer. Patients who undergo earlier surgical intervention tend to achieve superior outcomes (Nakamura et al., 2011). The younger the patient, the better the chance of recovery (Nakamura et al., 2011). Patients with ventrolateral dura defects have a significantly better prognosis than those with ventral dura defects (lateral vs central) (Nakamura et al., 2011; Nakashima et al., 2018). Additionally, the chosen surgical technique can influence success (Groen et al., 2019).

While our study revealed no significant differences in mean age and disease duration at the time of surgery between the two groups, a potential limitation is that preoperative myelopathy was not considered a confounding factor, which could have influenced our results. Furthermore, the initial symptomatology of patients could influence the outcome (Sasani et al., 2009), but this was not considered in our study. Additionally, the surgical outcome can be confounded by surgical complications. As depicted in Table 2, complications influenced the outcome. An additional limitation of this study is the inability to fully account for several potentially important confounding factors inherent to the retrospective, literature-based design. Variables such as the use and modality of intraoperative neuromonitoring, the presence of dural duplication or other anatomical variations, and the surgeon's discretion in selecting the operative technique were inconsistently reported. They could therefore not be systematically controlled for in the analysis. These unmeasured or incompletely reported factors may have influenced both the choice of surgical strategy and postoperative outcomes, thereby limiting causal interpretation of the observed associations. A key advantage of our study is the strict inclusion criteria for idiopathic spinal cord herniations, which enabled ISCH to be considered a distinct entity among spinal cord herniations and ensured study homogeneity. Furthermore, the large cohort size is another advantage.

## 5 Conclusion

Both ventral dural closure and non-closure (dural widening) strategies were associated with favourable clinical outcomes in the surgical management of idiopathic spinal cord herniation. In this literature-based analysis, the non-closure approach was associated with a higher likelihood of symptom improvement and comparable recurrence and complication rates; however, these findings should be interpreted as descriptive associations rather than evidence of superiority. Intraoperative neuromonitoring was frequently used and represents a valuable adjunct to guide the extent of spinal cord manipulation and to detect functional compromise early. In selected cases, deterioration of neuromonitoring signals may reasonably prompt avoidance or abandonment of dural closure, with a non-closure strategy constituting an acceptable alternative. Overall, these observations support hypothesis generation and underscore the need for individualised intraoperative decision-making, while prospective studies are required to define the comparative effectiveness of these techniques better.

## Authors’ contributions

UE, as the first author, conceived the study, contributed to data extraction, created the data list, participated in the calculation of data, and wrote the manuscript. ZO, SH, and SK reviewed the manuscript. AM, as the senior author, conceived the study, contributed to data extraction, performed the statistical analysis, reviewed the data list and manuscript. All authors approved the final version of the manuscript.

## Declaration of competing interest

The authors declare that they have **no known competing financial interests or personal relationships** that could have appeared to influence the work reported in this paper.
