# Supplementary material for: Surgical Treatment of Idiopathic spinal cord herniation: A Case Report under Neuromonitoring and Meta-analysis of 211 reviewed Cases
Source: Brain Spine. 2026 Feb 2;6:105958. doi: 10.1016/j.bas.2026.105958 (PMC12914459; doi:10.1016/j.bas.2026.105958)
Supplement: Multimedia component 1 [file mmc1.docx]

**References of included studies in the meta-analysis**

1. Nakazawa H, Toyama Y, Satomi K, Fujimura Y, Hirabayashi K. Idiopathic spinal

cord herniation. Report of two cases and review of the literature. Spine (Phila

Pa 1976). 1993 Oct 15;18(14):2138-41. PMID: 8272972.

1. Kumar R, Taha J, Greiner AL. Herniation of the spinal cord. Case report. J

Neurosurg. 1995 Jan;82(1):131-6. doi: 10.3171/jns.1995.82.1.0131. PMID: 7815118.

1. Borges LF, Zervas NT, Lehrich JR. Idiopathic spinal cord herniation: a

treatable cause of the Brown-Sequard syndrome--case report. Neurosurgery. 1995

May;36(5):1028-32; discussion 1032-3. doi: 10.1227/00006123-199505000-00023.

PMID: 7791969.

1. Miura Y, Mimatsu K, Matsuyama Y, Yoneda M, Iwata H. Idiopathic spinal cord

herniation. Neuroradiology. 1996 Feb;38(2):155-6. doi: 10.1007/BF00604805. PMID:

8692428.

1. Sioutos P, Arbit E, Tsairis P, Gargan R. Spontaneous thoracic spinal cord

herniation. A case report. Spine (Phila Pa 1976). 1996 Jul 15;21(14):1710-3.

doi: 10.1097/00007632-199607150-00019. PMID: 8839477.

1. Uchino A, Kato A, Momozaki N, Yukitake M, Kudo S. Spinal cord herniation:

report of two cases and review of the literature. Eur Radiol. 1997;7(2):289-92.

doi: 10.1007/s003300050153. PMID: 9038133.

1. Baur A, Stäbler A, Psenner K, Hamburger C, Reiser M. Imaging findings in

patients with ventral dural defects and herniation of neural tissue. Eur Radiol.

1997;7(8):1259-63. doi: 10.1007/s003300050286. PMID: 9377512.

1. Henry A, Tunkel R, Arbit E, Ku A, Lachmann E. Tethered thoracic cord

resulting from spinal cord herniation. Arch Phys Med Rehabil. 1997

May;78(5):530-3. doi: 10.1016/s0003-9993(97)90170-2. PMID: 9161375.

1. Miyake S, Tamaki N, Nagashima T, Kurata H, Eguchi T, Kimura H. Idiopathic

spinal cord herniation. Report of two cases and review of the literature. J

Neurosurg. 1998 Feb;88(2):331-5. doi: 10.3171/jns.1998.88.2.0331. PMID: 9452246.

1. Watters MR, Stears JC, Osborn AG, Turner GE, Burton BS, Lillehei K, Yuh WT.

Transdural spinal cord herniation: imaging and clinical spectra. AJNR Am J

Neuroradiol. 1998 Aug;19(7):1337-44. PMID: 9726479; PMCID: PMC8332200.

1. Dix JE, Griffitt W, Yates C, Johnson B. Spontaneous thoracic spinal cord

herniation through an anterior dural defect. AJNR Am J Neuroradiol. 1998

Aug;19(7):1345-8. PMID: 9726480; PMCID: PMC8332207.

1. Vallée B, Mercier P, Menei P, Bouhour F, Fischer C, Fournier D, Bougeard R,

Diabira S, Mahla K. Ventral transdural herniation of the thoracic spinal cord:

surgical treatment in four cases and review of literature. Acta Neurochir

(Wien). 1999;141(9):907-13. doi: 10.1007/s007010050396. PMID: 10526071.

1. Abe M, Komori H, Yamaura I, Kayano T. Spinal cord herniation into an

extensive extradural meningeal cyst: postoperative analysis of intracystic flow

by phase-contrast cine MRI. J Orthop Sci. 1999;4(6):450-6. doi:

10.1007/s007760050129. PMID: 10664429.

17. Marshman LA, Hardwidge C, Ford-Dunn SC, Olney JS. Idiopathic spinal cord

herniation: case report and review of the literature. Neurosurgery. 1999

May;44(5):1129-33. doi: 10.1097/00006123-199905000-00112. PMID: 10232549.

1. Brugières P, Malapert D, Adle-Biassette H, Fuerxer F, Djindjian M, Gaston A.

Idiopathic spinal cord herniation: value of MR phase-contrast imaging. AJNR Am J

Neuroradiol. 1999 May;20(5):935-9. PMID: 10369369; PMCID: PMC7056135

1. Tekkök IH. Spontaneous spinal cord herniation: case report and review of the

literature. Neurosurgery. 2000 Feb;46(2):485-91; discussion 491-2. doi:

10.1097/00006123-200002000-00044. PMID: 10690740.

17. Ewald C, Kühne D, Hassler WE. Progressive spontaneous herniation of the

thoracic spinal cord: case report. Neurosurgery. 2000 Feb;46(2):493-5;

discussion 495-6. doi: 10.1097/00006123-200002000-00046. PMID: 10690741.

1. Wada E, Yonenobu K, Kang J. Idiopathic spinal cord herniation: report of

three cases and review of the literature. Spine (Phila Pa 1976). 2000 Aug

1;25(15):1984-8. doi: 10.1097/00007632-200008010-00019. PMID: 10908944.

1. Pereira P, Duarte F, Lamas R, Vaz R. Idiopathic spinal cord herniation: case

report and literature review. Acta Neurochir (Wien). 2001;143(4):401-6. doi:

10.1007/s007010170096. PMID: 11437295.

20. Morokoff AP, Tress BM, Kaye AH. Idiopathic spinal cord herniation. J Clin

Neurosci. 2001 Mar;8(2):180-3. doi: 10.1054/jocn.2000.0819. PMID: 11243773.

1. Miyaguchi M, Nakamura H, Shakudo M, Inoue Y, Yamano Y. Idiopathic spinal

cord herniation asociated with intervertebral disc extrusion: a case report and

review of the literature. Spine (Phila Pa 1976). 2001 May 1;26(9):1090-4. doi:

10.1097/00007632-200105010-00022. PMID: 11337631.

22. Watanabe M, Chiba K, Matsumoto M, Maruiwa H, Fujimura Y, Toyama Y. Surgical

management of idiopathic spinal cord herniation: a review of nine cases treated

by the enlargement of the dural defect. J Neurosurg. 2001 Oct;95(2

Suppl):169-72. doi: 10.3171/spi.2001.95.2.0169. PMID: 11599832.

1. Eguchi T, Yokota H, Nikaido Y, Nobayashi M, Nishioka T. Spontaneous thoracic spinal cord herniation--case report. Neurol Med Chir (Tokyo). 2001

Oct;41(10):508-12. doi: 10.2176/nmc.41.508. PMID: 11760388.

1. Aizawa T, Sato T, Tanaka Y, Kotajima S, Sekiya M, Kokubun S. Idiopathic

herniation of the thoracic spinal cord: report of three cases. Spine (Phila Pa

1976). 2001 Oct 15;26(20):E488-91. doi: 10.1097/00007632-200110150-00030. PMID:

11598531.

1. Cellerini M, Bayon S, Scazzeri F, Mangiafico S, Amantini A, Guizzardi GC,

Giordano GP. Idiopatic spinal cord herniation: a treatable cause of Brown-

Séquard syndrome. Acta Neurochir (Wien). 2002 Apr;144(4):321-5. doi:

10.1007/s007010200044. PMID: 12021877.

26. Massicotte EM, Montanera W, Ross Fleming JF, Tucker WS, Willinsky R,

TerBrugge K, Fehlings MG. Idiopathic spinal cord herniation: report of eight

cases and review of the literature. Spine (Phila Pa 1976). 2002 May

1;27(9):E233-41. doi: 10.1097/00007632-200205010-00025. PMID: 11979181.

1. Barbagallo GM, Marshman LA, Hardwidge C, Gullan RW. Thoracic idiopathic

spinal cord herniation at the vertebral body level: a subgroup with a poor

prognosis? Case reports and review of the literature. J Neurosurg. 2002 Oct;97(3

Suppl):369-74. doi: 10.3171/spi.2002.97.3.0369. PMID: 12408396.

1. Iyer RV, Coutinho C, Lye RH. Spontaneous spinal cord herniation. Br J

Neurosurg. 2002 Oct;16(5):507-10. doi: 10.1080/0268869021000030960. PMID:

12498498.

1. Inoue T, Cohen-Gadol AA, Krauss WE. Low-pressure headaches and spinal cord

herniation. Case report. J Neurosurg. 2003 Jan;98(1 Suppl):93-5. doi:

10.3171/spi.2003.98.1.0093. PMID: 12546398.

30. Sasaoka R, Nakamura H, Yamano Y. Idiopathic spinal cord herniation in the

thoracic spine as a cause of intractable leg pain: case report and review of the

literature. J Spinal Disord Tech. 2003 Jun;16(3):288-94. doi:

10.1097/00024720-200306000-00011. PMID: 12792345.

31. Saito T, Anamizu Y, Nakamura K, Seichi A. Case of idiopathic thoracic spinal

cord herniation with a chronic history: a case report and review of the

literature. J Orthop Sci. 2004;9(1):94-8. doi: 10.1007/s00776-003-0730-y. PMID:

14767711.

32. White BD, Tsegaye M. Idiopathic anterior spinal cord hernia: under-

recognized cause of thoracic myelopathy. Br J Neurosurg. 2004 Jun;18(3):246-9.

doi: 10.1080/02688690410001732670. PMID: 15327225.

33. Gwinn R, Henderson F. Transdural herniation of the thoracic spinal cord:

untethering via a posterolateral transpedicular approach. Report of three cases.

J Neurosurg Spine. 2004 Sep;1(2):223-7. doi: 10.3171/spi.2004.1.2.0223. PMID:

15347010.

34. Sugimoto T, Kasai Y, Takegami K, Morimoto R, Maeda M, Uchida A. A case of

idiopathic spinal cord herniation with duplicated dura mater. J Spinal Disord

Tech. 2005 Feb;18(1):106-11. doi: 10.1097/01.bsd.0000123427.12852.ae. PMID:

15687862.

35. Ferré JC, Carsin-Nicol B, Hamlat A, Carsin M, Morandi X. MR imaging features

of idiopathic thoracic spinal cord herniations using combined 3D-fiesta and

2D-PC Cine techniques. J Neuroradiol. 2005 Mar;32(2):125-30. doi:

10.1016/s0150-9861(05)83127-x. PMID: 15984404.

36. Karadeniz-Bilgili MY, Castillo M, Bernard E. Transdural spinal cord

herniation: pre- and postoperative MRI findings. Clin Imaging. 2005 Jul-

Aug;29(4):288-90. doi: 10.1016/j.clinimag.2004.09.001. PMID: 15967323.

37. Ammar KN, Pritchard PR, Matz PG, Hadley MN. Spontaneous thoracic spinal cord

herniation: three cases with long-term follow-up. Neurosurgery. 2005

Nov;57(5):E1067; discussion E1067. doi: 10.1227/01.neu.0000180016.69507.e0.

PMID: 16284547.

38. Maira G, Denaro L, Doglietto F, Mangiola A, Colosimo C. Idiopathic spinal

cord herniation: diagnostic, surgical, and follow-up data obtained in five

cases. J Neurosurg Spine. 2006 Jan;4(1):10-9. doi: 10.3171/spi.2006.4.1.10.

PMID: 16506460.

39. Morley S, Naidoo P, Robertson A, Chong W. Thoracic ventral dural defect:

idiopathic spinal cord herniation. Australas Radiol. 2006 Apr;50(2):168-70. doi:

10.1111/j.1440-1673.2006.01547.x. PMID: 16635036.

40. Saito A, Takahashi T, Sato S, Kumabe T, Tominaga T. Modified surgical

technique for the treatment of idiopathic spinal cord herniation. Minim Invasive

Neurosurg. 2006 Apr;49(2):120-3. doi: 10.1055/s-2006-932171. PMID: 16708343.

41. Ellger T, Schul C, Heindel W, Evers S, Ringelstein EB. Idiopathic spinal

cord herniation causing progressive Brown-Séquard syndrome. Clin Neurol

Neurosurg. 2006 Jun;108(4):388-91. doi: 10.1016/j.clineuro.2004.07.005. Epub

2006 Feb 17. PMID: 16483712.

42. Darbar A, Krishnamurthy S, Holsapple JW, Hodge CJ Jr. Ventral thoracic

spinal cord herniation: frequently misdiagnosed entity. Spine (Phila Pa 1976).

2006 Aug 1;31(17):E600-5. doi: 10.1097/01.brs.0000229247.69171.a1. PMID:

16924199.

43. Arts MP, Lycklama à Nijeholt G, Wurzer JA. Surgical treatment of idiopathic

transdural spinal cord herniation: a new technique to untether the spinal cord.

Acta Neurochir (Wien). 2006 Sep;148(9):1005-9. doi: 10.1007/s00701-006-0783-8.

Epub 2006 Jun 12. PMID: 16770516.

44. Barrenechea IJ, Lesser JB, Gidekel AL, Turjanski L, Perin NI. Diagnosis and

treatment of spinal cord herniation: a combined experience. J Neurosurg Spine.

2006 Oct;5(4):294-302. doi: 10.3171/spi.2006.5.4.294. PMID: 17048765.

45. Kim JM, Oh SH, Kim KJ, Park SH, Park KS. Idiopathic spinal cord herniation

as a treatable cause of progressive brown-sequard syndrome. J Clin Neurol. 2007

Dec;3(4):204-7. doi: 10.3988/jcn.2007.3.4.204. Epub 2007 Dec 20. PMID: 19513134;

PMCID: PMC2686945.

46. Uhl E, Holtmannspötter M, Tonn JC. Improvement of Brown-Sequard syndrome

after surgical repair of an idiopathic thoracic spinal cord herniation. J

Neurol. 2008 Jan;255(1):125-6. doi: 10.1007/s00415-008-0553-z. Epub 2008 Jan 22.

PMID: 18204810.

47. Ishida M, Maeda M, Kasai Y, Uchida A, Takeda K. Idiopathic spinal cord

herniation through the inner layer of duplicated anterior dura: evaluation with

high-resolution 3D MRI. J Clin Neurosci. 2008 Aug;15(8):933-7. doi:

10.1016/j.jocn.2006.10.024. Epub 2008 May 22. PMID: 18501612.

48. Sasani M, Ozer AF, Vural M, Sarioglu AC. Idiopathic spinal cord herniation:

case report and review of the literature. J Spinal Cord Med. 2009;32(1):86-94.

doi: 10.1080/10790268.2009.11760757. PMID: 19264054; PMCID: PMC2647506.

49. Chaichana KL, Sciubba DM, Li KW, Gokaslan ZL. Surgical management of

thoracic spinal cord herniation: technical consideration. J Spinal Disord Tech.

2009 Feb;22(1):67-72. doi: 10.1097/BSD.0b013e318165fe2a. PMID: 19190439.

50. Ghostine S, Baron EM, Perri B, Jacobson P, Morsette D, Hsu FP. Thoracic

cord herniation through a dural defect: description of a case and review of the

literature. Surg Neurol. 2009 Mar;71(3):362-6, discussion 366-7. doi:

10.1016/j.surneu.2007.08.022. Epub 2008 Jan 22. PMID: 18207514.

51. Selviaridis P, Balogiannis I, Foroglou N, Hatzisotiriou A, Patsalas I.

Spontaneous spinal cord herniation: recurrence after 10 years. Spine J. 2009

Mar;9(3):e17-9. doi: 10.1016/j.spinee.2008.03.013. Epub 2008 Jun 6. PMID:

18538639.

52. Groen RJ, Middel B, Meilof JF, de Vos-van de Biezenbos JB, Enting RH,

Coppes MH, Journee LH. Operative treatment of anterior thoracic spinal cord

herniation: three new cases and an individual patient data meta-analysis of 126

case reports. Neurosurgery. 2009 Mar;64(3 Suppl):ons145-59; discussion

ons159-60. doi: 10.1227/01.NEU.0000327686.99072.E7. PMID: 19240564.

53. Sai Kiran NA, Vaishya S, Kasliwal MK, Kale SS, Sharma BS. Spontaneous

thoracic spinal cord herniation presenting as tethered cord syndrome. Neurol

India. 2009 Mar-Apr;57(2):221-2. doi: 10.4103/0028-3886.51306. PMID: 19439866.

54. Imagama S, Matsuyama Y, Sakai Y, Nakamura H, Katayama Y, Ito Z, Wakao N,

Sato K, Kamiya M, Kato F, Yukawa Y, Miura Y, Yoshihara H, Suzuki K, Ando K,

Hirano K, Tauchi R, Muramoto A, Ishiguro N. Image classification of idiopathic

spinal cord herniation based on symptom severity and surgical outcome: a

multicenter study. J Neurosurg Spine. 2009 Sep;11(3):310-9. doi:

10.3171/2009.4.SPINE08691. PMID: 19769512.

55. Fallah A, Fehlings MG. Congenital ventral thoracic spinal cord herniation.

Can J Neurol Sci. 2010 Mar;37(2):271-2. doi: 10.1017/s0317167100010052. PMID:

20437942.

56. Zairi F, Thines L, Bourgeois P, Dereeper O, Assaker R. Spinal cord

herniation: a misdiagnosed and treatable cause of thoracic myelopathy. Acta

Neurochir (Wien). 2010 Nov;152(11):1991-6. doi: 10.1007/s00701-010-0773-8. Epub

2010 Aug 21. PMID: 20730456.

57. Nakamura M, Fujiyoshi K, Tsuji O, Watanabe K, Tsuji T, Ishii K, Matsumoto

M, Toyama Y, Chiba K. Long-term surgical outcomes of idiopathic spinal cord

herniation. J Orthop Sci. 2011 Jul;16(4):347-51. doi: 10.1007/s00776-011-0065-z.

Epub 2011 May 5. PMID: 21544598; PMCID: PMC3140945.

58. Aydin AL, Sasani M, Erhan B, Sasani H, Ozcan S, Ozer AF. Idiopathic spinal

cord herniation at two separate zones of the thoracic spine: the first reported

case and literature review. Spine J. 2011 Aug;11(8):e9-e14. doi:

10.1016/j.spinee.2011.07.003. Epub 2011 Sep 8. PMID: 21862417.

59. Akutsu H, Takada T, Nakai K, Tsuda K, Sakane M, Aita I, Matsumura A.

Surgical technique for idiopathic spinal cord herniation: the Hammock method.

Technical note. Neurol Med Chir (Tokyo). 2012;52(4):238-42. doi:

10.2176/nmc.52.238. PMID: 22522340.

60. Novak K, Widhalm G, de Camargo AB, Perin N, Jallo G, Knosp E, Deletis V.

The value of intraoperative motor evoked potential monitoring during surgical

intervention for thoracic idiopathic spinal cord herniation. J Neurosurg Spine.

2012 Feb;16(2):114-26. doi: 10.3171/2011.10.SPINE11109. Epub 2011 Nov 25. PMID:

22117142.

61. Kasliwal MK, O'toole JE, Deutsch H. Unilateral paramedian transpedicular

approach for repair of anterior transdural spinal cord herniation: report of a

case and literature review. Asian Spine J. 2012 Mar;6(1):55-9. doi:

10.4184/asj.2012.6.1.55. Epub 2012 Mar 9. PMID: 22439089; PMCID: PMC3302916.

62. Prada F, Saladino A, Giombini S, Erbetta A, Saini M, DiMeco F, Lodrini S.

Spinal cord herniation: management and outcome in a series of 12 consecutive

patients and review of the literature. Acta Neurochir (Wien). 2012

Apr;154(4):723-30. doi: 10.1007/s00701-011-1265-1. PMID: 22290791.

63. Goetti R, Wille D, Kretzschmar U, Klein A, Scheer I. Idiopathic spinal cord

herniation: first reported case in a child. JAMA Neurol. 2013 Jan;70(1):125-6.

doi: 10.1001/jamaneurol.2013.586. PMID: 23070462.

64. Prasad A, Brar R, Sinha S, Rana S. Idiopathic spinal cord herniation.

Singapore Med J. 2013 Feb;54(2):e43-5. PMID: 23462842.

65. Berg-Johnsen J, Ilstad E, Kolstad F, Züchner M, Sundseth J. Idiopathic

ventral spinal cord herniation: an increasingly recognized cause of thoracic

myelopathy. J Cent Nerv Syst Dis. 2014 Oct 1;6:85-91. doi: 10.4137/JCNSD.S16180.

PMID: 25336997; PMCID: PMC4196882.

66. Yamamoto N, Katoh S, Higashino K, Sairyo K. Idiopathic spinal cord

herniation with duplicated dura mater and dorsal subarachnoid septum. Report of

a case and review of the literature. Int J Spine Surg. 2014 Dec 1;8:29. doi:

10.14444/1029. PMID: 25694934; PMCID: PMC4325502.

67. De Souza RB, De Aguiar GB, Daniel JW, Veiga JC. The pathophysiology,

classification, treatment, and prognosis of a spontaneous thoracic spinal cord

herniation: A case study with literature review. Surg Neurol Int. 2014 Dec

30;5(Suppl 15):S564-6. doi: 10.4103/2152-7806.148042. PMID: 25593778; PMCID:

PMC4287899.

68. Martinez-del-Campo E, Moon K, Kalb S, Soriano-Baron H, Theodore N. Surgical

Management of a Patient With Thoracic Spinal Cord Herniation: Case Report.

Neurosurgery. 2015 Sep;77(3):E492-8; discussion E498-9. doi:

10.1227/NEU.0000000000000860. PMID: 26110998.

69. Ju MW, Choi SW, Youm JY, Kwon HJ. Idiopathic Spinal Cord Herniation

Presented as Brown-Sequard Syndrome : A Case Report and Surgical Outcome. J

Korean Neurosurg Soc. 2015 Sep;58(3):294-7. doi: 10.3340/jkns.2015.58.3.294.

Epub 2015 Sep 30. PMID: 26539277; PMCID: PMC4630365.

70. Payer M, Zumsteg D, De Tribolet N, Wetzel S. Surgical management of

thoracic idiopathic spinal cord herniation. Technical case report and review.

Acta Neurochir (Wien). 2016 Aug;158(8):1579-82. doi: 10.1007/s00701-016-2840-2.

Epub 2016 May 25. PMID: 27221089.

71. Alkhamees A, Proust F. Idiopathic Spinal Cord Herniation: A Case Report.

Int J Health Sci (Qassim). 2016 Oct;10(4):592-595. PMID: 27833524; PMCID:

PMC5085354.

72. Delgado-López PD, Gil-Polo C, Martín-Velasco V, Martín-Alonso J, Galacho-

Harriero AM, Araus-Galdós E. Spinal cord herniation repair with microstaples:

case report. J Neurosurg Spine. 2017 Mar;26(3):384-387. doi:

10.3171/2016.8.SPINE16318. Epub 2016 Nov 4. PMID: 27813449.

73. Reddy R, Farha F, Babu P. Idiopathic ventral thoracic spinal cord

herniation with duplicated dura mater and subarachnoid septum. Neurol India.

2017 Jul-Aug;65(4):906-908. doi: 10.4103/neuroindia.NI_1064_16. PMID: 28681783.

74. Bartels RHMA, Brunner H, Hosman A, van Alfen N, Grotenhuis JA. The

Pathogenesis of Ventral Idiopathic Herniation of the Spinal Cord: A Hypothesis

Based on the Review of the Literature. Front Neurol. 2017 Sep 11;8:476. doi:

10.3389/fneur.2017.00476. PMID: 28955299; PMCID: PMC5601982.

75. Gkekas N, Kasapas K, Sioutos P, Georgakoulias N. Duplication of the dura as

a cause of anterior thoracic spinal cord herniation. A case report. Br J

Neurosurg. 2017 Oct;31(5):616-618. doi: 10.1080/02688697.2016.1199785. Epub 2016

Jun 22. PMID: 27331899.

76. Florian B, Luc LF, Philippe M, Jean-Michel L, Fournier HD. Transdural

Spinal Cord Herniation: Tips and Tricks. World Neurosurg. 2018 Jan;109:242-246.

doi: 10.1016/j.wneu.2017.09.195. Epub 2017 Oct 7. PMID: 29017979.

77. Ghali MGZ, Srinivasan VM, Rao VY, Omeis I. Idiopathic thoracic spinal cord

herniation. J Clin Neurosci. 2018 May;51:1-5. doi: 10.1016/j.jocn.2017.10.090.

Epub 2018 Feb 19. PMID: 29472069.

78. Shimizu S, Kobayashi Y, Oka H, Kumabe T. Idiopathic spinal cord herniation:

consideration of its pathogenesis based on the histopathology of the dura mater.

Eur Spine J. 2019 Feb;28(2):298-305. doi: 10.1007/s00586-017-5147-y. Epub 2017

Jun 7. PMID: 28593382.

79. Hlubek RJ, Xu DS, Mulholland CB, Gilson J, Theodore N, Turner JD, Kakarla

UK. Operative Management of Idiopathic Spinal Cord Herniation: Case Series and

Novel Technique for Repair of Recurrent Herniation. Oper Neurosurg (Hagerstown).

2019 Apr 1;16(4):415-423. doi: 10.1093/ons/opy129. PMID: 30011010.

80. Gomez-Amarillo D, Garcia-Baena C, Volcinschi-Moros D, Hakim F. Thoracic

idiopathic spinal cord herniation in a young patient: a diagnostic and

therapeutic challenge. BMJ Case Rep. 2019 May 21;12(5):e227847. doi:

10.1136/bcr-2018-227847. PMID: 31118170; PMCID: PMC6559815.

81. Tyagi G, A R P, Bhat DI, Rao MB, Devi BI. Duplication of Ventral Dura as a

Cause of Ventral Herniation of Spinal Cord-A Report of Two Cases and Review of

the Literature. World Neurosurg. 2019 Jun;126:346-353. doi:

10.1016/j.wneu.2019.02.143. Epub 2019 Mar 6. PMID: 30851464.

82. Herring EZ, Shin JH, Nagel SJ, Krishnaney AA. Novel Strategy of Ventral

Dural Repair for Idiopathic Thoracic Spinal Cord Herniation: Report of Outcomes

and Review of Techniques. Oper Neurosurg (Hagerstown). 2019 Jul 1;17(1):21-31.

doi: 10.1093/ons/opy244. PMID: 30517700.

83. Iunes EA, Barletta EA, Suzuki FS, Barba Belsuzarri TA, de Araújo Paz D,

Veiga de Castro Sparapani F, Onishi FJ, Cavalheiro S, Salati T, de Meldau

Benites V, Riechelmann G, Joaquim AF. Idiopathic Ventral Spinal Cord Herniation:

Video Report and Systematic Review. World Neurosurg. 2020 Jul;139:592-602. doi:

10.1016/j.wneu.2020.04.190. Epub 2020 May 4. PMID: 32376383.

84. Regensburger M, Schlachetzki JCM, Klekamp J, Doerfler A, Winkler J. Long-

term course of anterior spinal cord herniation presenting with an upper motor

neuron syndrome: case report illustrating diagnostic and therapeutic

implications. BMC Neurol. 2020 Aug 29;20(1):321. doi:

10.1186/s12883-020-01891-1. PMID: 32861240; PMCID: PMC7455782.

85. Dogu H, Ozdemir NG, Yilmaz H, Turk O, Demirel N, Atci IB, Kocak A. A Rare

Entity: Idiopathic Spinal Cord Herniation. Neurol India. 2020 Nov-

Dec;68(6):1472-1474. doi: 10.4103/0028-3886.304129. PMID: 33342896.

86. Teng KX, Dimou J. Delayed cord tethering post-ventral dural repair of

idiopathic thoracic cord herniation. J Clin Neurosci. 2021 Jun;88:1-4. doi:

10.1016/j.jocn.2021.02.026. Epub 2021 Mar 29. PMID: 33992165.

87. Ohtake Y, Senoo M, Fukuda M, Ishida Y, Yoshihara R, Ishikawa K, Fuchizaki

T, Ishizuka T, Okamura N, Nakamura H. Symptomatic interdural cerebrospinal fluid

collections after surgery for idiopathic spinal cord herniation: illustrative

case. J Neurosurg Case Lessons. 2021 Oct 4;2(14):CASE21232. doi:

10.3171/CASE21232. PMID: 36131572; PMCID: PMC9563952.

88. Vanden Bulcke D, Baussart B, Auliac S, Boulin A, Gaillard S. A unique case

of pure lateral spinal cord herniation. Neurochirurgie. 2021 Nov;67(6):624-627.

doi: 10.1016/j.neuchi.2020.12.006. Epub 2021 Jan 12. PMID: 33450267.

89. Yang C, Lin G, Zhang J, Yang J, Xie J. Case Report: Idiopathic Spinal Cord

Herniation: An Overlooked and Frequently Misdiagnosed Entity. Front Surg. 2022

May 20;9:905038. doi: 10.3389/fsurg.2022.905038. PMID: 35711698; PMCID:

PMC9195413.

90. Fischer G, Kälin V, Gautschi OP, Bozinov O, Stienen MN. Expanding the

indications for measurement of objective functional impairment in spine surgery:

A pilot study of four patients with diseases affecting the spinal cord. Brain

Spine. 2022 Jul 20;2:100915. doi: 10.1016/j.bas.2022.100915. PMID: 36248131;

PMCID: PMC9560588.

91. Zhang L, Wu H, Liu Z, Wang X, Cheng Y, Wang K. Dural repair with fat patch

for idiopathic spinal cord herniation: operative technique and a review of seven

cases. Ann Transl Med. 2022 Aug;10(16):865. doi: 10.21037/atm-22-3343. PMID:

36111002; PMCID: PMC9469156.

92. Kamamura M, Higaki F, Sasada S, Matsushita T, Yasuhara T, Date I, Hiraki T.

A Rare Case of Idiopathic Spinal Cord Herniation Treated by DuraGen® Collagen

Matrix Graft. Acta Med Okayama. 2022 Dec;76(6):731-736. doi: 10.18926/AMO/64124.

PMID: 36549776.

93. Farrokhi MR, Mousavi SR, Rafieossadat R. Idiopathic spinal cord herniation

at the cervicothoracic junction level presenting with unilateral sensory

symptoms. Clin Neurol Neurosurg. 2023 Jan;224:107526. doi:

10.1016/j.clineuro.2022.107526. Epub 2022 Nov 15. PMID: 36442311.

94. Hajiabadi M, Pirhadi M, Goudarzi Taemeh D, Amirjamshidi A. Remarkable

improvement of neurological deficits after surgery in patients with Idiopathic

spinal cord herniations. The impact of peroperative neuromonitoring. Case

reports. Brain Spine. 2023 Jul 21;3:101785. doi: 10.1016/j.bas.2023.101785.

PMID: 38021003; PMCID: PMC10668057.

95. Jiang Q, Gao G, Tao B, Gao H, Wang H, Wang P, Sun M, Shang A. Thoracic

Anterior Spinal Cord Herniation: Treatment and Prognosis Outcome of Seven

Patients. World Neurosurg. 2023 Aug;176:e697-e703. doi:

10.1016/j.wneu.2023.06.001. Epub 2023 Jun 8. PMID: 37295472.

96. Hirose Y, Nagoshi N, Tsuji O, Kono H, Iida T, Suzuki S, Takahashi Y, Nori

S, Yagi M, Matsumoto M, Nakamura M, Watanabe K. Natural history and surgical

outcomes of idiopathic spinal cord herniation. Spinal Cord. 2023

Aug;61(8):441-446. doi: 10.1038/s41393-023-00904-3. Epub 2023 Jun 28. PMID:

37380759.

97. Kaida M, Hirata H, Noda H, Kishikawa Y, Yoshihara T, Kobayashi T, Tsukamoto

M, Mawatari M, Morimoto T. Early detection of idiopathic thoracic ventral spinal

cord herniation by using imaging: A case report. Clin Case Rep. 2023 Nov

9;11(11):e8112. doi: 10.1002/ccr3.8112. PMID: 37953892; PMCID: PMC10636536.

98. Ifthekar S, Shin SH, Lee SH, Bae J. Idiopathic spinal cord herniation with

postoperative paraplegia-A case report. Clin Case Rep. 2023 Dec 17;11(12):e8246.

doi: 10.1002/ccr3.8246. PMID: 38111513; PMCID: PMC10726001.

99. Masuzawa H, Nakayama H, Shitara N, Suzuki T. Spinal cord herniation into a

congenital extradural arachnoid cyst causing Brown-Séquard syndrome. Case

report. J Neurosurg. 1981 Dec;55(6):983-6. doi: 10.3171/jns.1981.55.6.0983.

PMID: 7299475.

100. Buntting CS, Ham Y, Teng KX, Dimou J, Gauden AJ, Nair G. Scalpel sign:

Dorsal thoracic arachnoid web, thoracic arachnoid cyst and ventral cord

herniation. Radiol Case Rep. 2022 Jul 28;17(10):3564-3569. doi:

10.1016/j.radcr.2022.06.100. PMID: 35923346; PMCID: PMC9340144.

101. Lui et al. Spinal Cord Suspension Using Dentate Ligament Hitch Stitches: A novel Technique for the Repair of Ventral Spinal Cord Herniation. Operative Neurosurgery14:252–258,2018. DOI:10.1093/ons/opx108.

102. L. Castellan et al. Spinal cord herniation. Available online 15 August 2008 doi:10.1016/j.neurad.2008.07.002.
